# Endoscopic-Assisted Adenoidectomy by Microdebrider Versus Coblation in Children

**DOI:** 10.3390/medicina62061048

**Published:** 2026-05-28

**Authors:** Hesham Abdelsalam, Mohamed Abdalla, Najlaa Aied, Sherif M. Elaidy, Osama Refaat, Mohamed Shams Eldin

**Affiliations:** 1 Department of Otorhinolaryngology, Faculty of Medicine, Al-Azhar University, Cairo 11675, Egypt; drhesham69ent@yahoo.com (H.A.); mohamedabdallaent@gmail.com (M.A.); osamarefaatent@yahoo.com (O.R.); 2 Department of Otorhinolaryngology, Prince Sultan Military Armed Forces Hospital, Riyadh 12233, Saudi Arabia; najlaaaiedent@gmail.com; 3 Department of Otorhinolaryngology, Faculty of Medicine, Al-Azhar University for Girls, Damietta 34517, Egypt; sherifelaidyent@gmail.com

**Keywords:** adenoidectomy, endoscopic, microdebrider, adenoid hypertrophy

## Abstract

*Background and Objectives*: Adenoidectomy is a common pediatric procedure performed and endoscopic, instrumented techniques such as a powered microdebrider and coblation aim to improve visualization and completeness of resection while potentially reducing pain, bleeding and recurrence compared with blind curettage. To compare clinical outcomes and safety of endoscopic-assisted adenoidectomy using a microdebrider versus coblation in pediatric patients. *Materials and Methods*: This retrospective comparative cohort study included 50 children who underwent endoscopic-assisted adenoidectomy using either a microdebrider (n = 25) or coblation (n = 25) during a 6-month study period. Operative time (skin-to-skin), pain scores at 24 h, early complications at prespecified intervals, and endoscopic assessment at 6 months were extracted from medical records and compared. *Results*: Groups were comparable at baseline (mean age ≈ 9.6 years, all baseline *p* > 0.05). Mean skin-to-skin operative time was significantly shorter with the microdebrider (24.9 ± 6.9 min) than with coblation (31.1 ± 8.4 min), *p* = 0.004. Mean 24 h VAS did not differ significantly between the microdebrider and coblation (3.20 ± 1.21 vs. 2.73 ± 1.30, *p* = 0.191). Early complication rates (postoperative bleeding within 48 h, infection within 7 d, halitosis, neck pain) were low and showed no statistically significant differences (all *p* > 0.05). *Conclusions*: Both microdebrider and coblation endoscopic adenoidectomy were safe and effective in this cohort study. The microdebrider was associated with a shorter operative time in our setting, while early pain, short-term complications and six-month endoscopic residual/recurrence were similar between techniques.

## 1. Introduction

Adenoid hypertrophy is a major cause of nasal obstruction, sleep-disordered breathing and recurrent otitis media in children and remains one of the most performed pediatric ENT operations worldwide. Surgical techniques have evolved from blind curettage to endoscopic-assisted techniques using powered instruments (microdebrider) and energy-based ablation (coblation) to improve completeness of resection and reduce morbidity [1,2,3].

Two commonly used endoscopic techniques are transoral microdebrider-assisted adenoidectomy and coblation-assisted adenoidectomy. Prior randomized and prospective studies have reported mixed findings: the microdebrider may offer a shorter operative time while coblation may reduce intraoperative bleeding and postoperative pain; however, heterogeneity in outcomes and limited resource-use data remain gaps in the literature [4,5].

These mixed results mean there is not a universal “best” technique for every outcome; technique choice is often guided by which outcome (speed, blood loss, postoperative pain, residual tissue and cost) is most important for a given patient or setting [6].

This study tested the hypothesis that coblation and microdebrider techniques differ in patient-centered recovery (pain, time to normal diet/activity) and resource metrics (operative time, disposables) and seeks to establish which technique optimizes the balance between completeness of removal, clinical recovery, and resource utilization.

### 1.1. Primary

1. Compare operative time (skin-to-skin, in minutes) between techniques.
2. Compare postoperative pain at 24 h using an age-appropriate 0–10 VAS (or FLACC for younger children).
3. Compare short-term postoperative complications within 48–72 h primary/secondary bleeding, surgical site infection, otitis media, halitosis, neck pain.

### 1.2. Secondary

1. Compare requirement for additional hemostasis (bipolar or other).
2. Assess adequacy of removal and endoscopic recurrence at 6 months.

## 2. Materials and Methods

### 2.1. Study Design

This was a retrospective, single-center, comparative cohort study of pediatric patients undergoing endoscopic transoral adenoidectomy using either a powered microdebrider or coblation. Consecutive eligible cases performed during the predefined 6-month study period were included. The choice of surgical technique was based on surgeon preference and/or device availability (non-randomized treatment modality). Standard postoperative follow-up in our unit included assessment at 24 h, 1 week, 1 month, and endoscopic assessment at 6 months. Clinical data were accessed for research purposes, extracted, reviewed, and analyzed between 15 October 2025 and 15 January 2026, during which patient file retrieval, data verification, and statistical analysis were completed.

### 2.2. Ethical Consideration

This study was conducted in accordance with the ethical principles outlined in the Declaration of Helsinki. The research was carried out at the Department of Otorhinolaryngology, Prince Sultan Military Armed Force Hospital, Madinah, Kingdom of Saudi Arabia. Ethical approval was obtained from the institutional review board/ethics committee (Approval No.: REC-25-24, dated 15 October 2025). As this was a retrospective review of routinely collected clinical data, the requirement for individual informed consent was waived by the ethics committee where applicable. Participant confidentiality and data privacy were strictly maintained throughout the study.

### 2.3. Sample Size and Power

This was a retrospective review of all eligible cases during the study period; therefore, no priori sample size calculation was performed. A total of 50 children met the inclusion criteria (25 treated with microdebrider and 25 treated with coblation).

### 2.4. Eligibility Criteria

The study included children aged 3–15 years with symptomatic adenoid hypertrophy presenting with nasal obstruction, recurrent otitis media, and/or symptoms suggestive of obstructive sleep-disordered breathing. Adenoid hypertrophy was confirmed by nasal endoscopy, with clinically significant disease defined as Parikh/Cassano grade ≥ 2 where endoscopic assessment was indicated. Eligible participants were candidates for adenoidectomy without a planned concurrent tonsillectomy, allowing postoperative outcomes (particularly pain) to be attributed solely to adenoidectomy. Children were excluded if they had a history of prior adenoid surgery, significant craniofacial or congenital anomalies (including cleft palate), known bleeding disorders or use of anticoagulant therapy, uncontrolled systemic illness, or an upper respiratory tract infection within two weeks prior to surgery. Patients whose parents or guardians declined consent or whose assent was not obtained, when applicable, were also excluded. The Parikh/Cassano endoscopic classification was used for baseline stratification and to document the completeness of adenoid resection as well as postoperative recurrence.

### 2.5. Data Collection

Data were extracted from the electronic medical record, operative notes, anesthesia records, and routine postoperative follow-up charts using a standardized data abstraction form. Endoscopic findings at 6 months were obtained from routine clinic endoscopy documentation.

### 2.6. Interventions—Operative Technique and Perioperative Standardization

General anesthesia with oral endotracheal intubation; patient supine; Boyle–Davis mouth gag; a 0° endoscope (rigid) used for visualization in all cases; all surgeons use a standardized approach and the same perioperative medications and intraoperative documentation procedures. All procedures were performed by a small group of experienced otolaryngologists at our institution, working within a standardized operative and perioperative protocol. Most cases in each arm were performed by the same senior surgeons.

### 2.7. Microdebrider Group

Endoscopic transoral microdebrider used in oscillating mode with continuous suction and curved blade appropriate for nasopharyngeal contour. Bipolar cautery allowed as needed for focal hemostasis (documented as “Hemostasis needed by bipolar” = yes/no and duration/number of applications recorded). Record microdebrider blade type/catalog, rpm setting, and suction pressure if relevant.

### 2.8. Coblation Group

- Endoscopic transoral coblation wand used as per manufacturer recommendations with recommended power and saline irrigation settings. Rinsing/clearing wand as required. Record coblation wand model, power settings, and number of passes.
- Operative time (minutes): defined as “knife (or instrument) in to instrument out”—your specified “skin-to-skin” or “instrument-to-instrument” is acceptable: start when the device first contacts adenoid tissue; stop when final hemostasis verified and scope removed. We emphasize that our measured time includes all stages from initial contact with adenoid tissue through final hemostasis and endoscope removal.
- Pain at 24 h: Age-appropriate scales:
  ○ Children ≥ 7 years: Numeric/visual analog scale 0–10 (VAS).
  ○ 3–6 years: Faces pain scale (or validated 0–10 conversion).
  ○ Younger children: FLACC scale recorded by trained assessor or parent proxy; convert to 0–10 analog for analysis.
- Short-term complications (within 48–72 h): binary outcomes—postoperative bleed (yes/no; graded as minor requiring only observation vs. major requiring return to theater), infection (clinical diagnosis with/without antibiotics), otitis media (new diagnosis requiring treatment), halitosis (parent/patient report or graded scale), neck pain (parent/patient report).
- Requirement for additional hemostasis: use of bipolar, number of applications/time (documented as binary + semi-quantitative).
- Analgesic consumption: total mg paracetamol-equivalents in first 24 h (convert any other analgesic to paracetamol-equivalents via prespecified conversion table).

### 2.9. Endoscopic 6-Month Assessment

At our center, routine 6-month follow-up nasoendoscopy is typically performed in the outpatient clinic by an ENT clinician who is not necessarily the operating surgeon. For this retrospective study, we extracted these routine endoscopic findings from the medical records, using the same Cassano/Parikh grading system to classify residual adenoid tissue as ‘none’, ‘small’, or ‘significant’.

### 2.10. Statistical Analysis

The results were arranged, tabulated, and statistically analyzed using the statistical package for social sciences (SPSS stat. 30) software, specifically version 11. For quantitative data, the mean and standard deviation were calculated, and Student’s *t*-test was used to statistically analyze the difference between two means. For qualitative data, the number and percentage of disturbances were approximated. The chi square test was used to determine significance, and Fisher’s exact test was used if it proved ineffectual. The significance level for interpreting the test results was established at *p* > 0.05.

## 3. Results

Baseline demographic and clinical characteristics were well balanced between the two study cohorts (Table 1). The mean age was 9.56 ± 3.99 years in the microdebrider group and 9.64 ± 3.84 years in the coblation group, with no statistically significant difference between groups (Mann–Whitney U test, *p* = 0.977). There were also no significant differences with respect to body weight, sex distribution, primary surgical indication, or preoperative adenoid hypertrophy grade (all *p* > 0.05).

**Figure 1:**
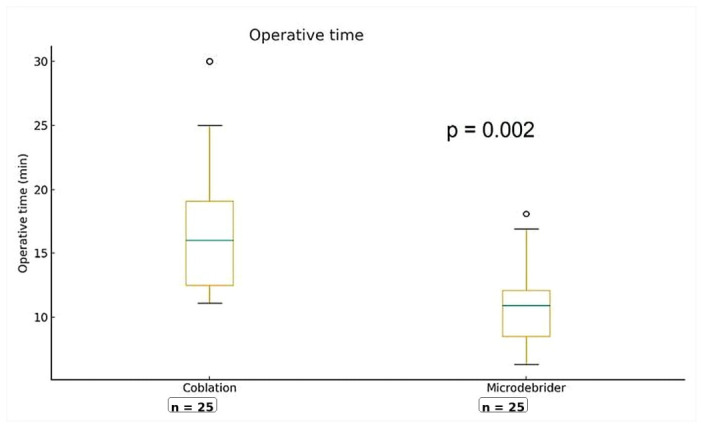
Comparison of operative time in both groups.

**Figure 2:**
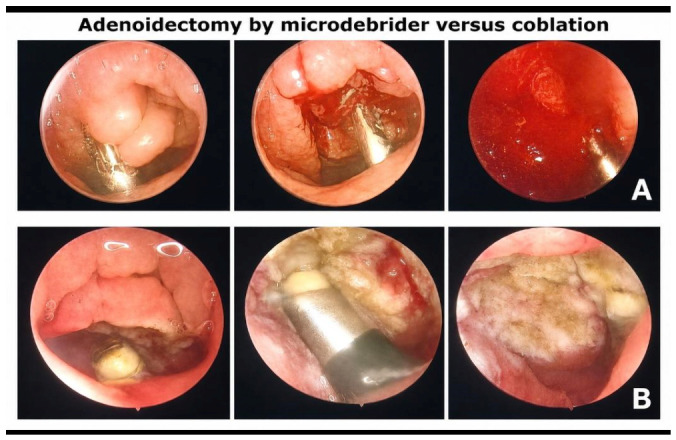
Adenoidectomy by microdebrider (**A**) versus coblation (**B**). Minimal bleeding occurred in some cases with microdebrider which was self-limited while almost no bleeding occurred using coblation as instant hemostasis was achieved by plasma generation.

**Figure 3:**
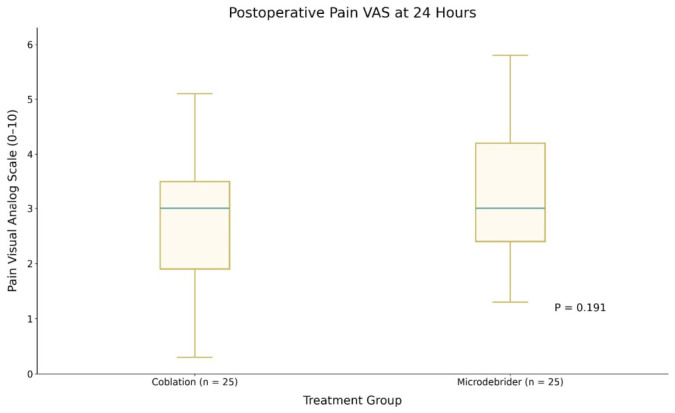
Comparison of operative pain (at 24 h) in both groups.

Total operative time (skin-to-skin) was significantly longer in the coblation arm compared with the microdebrider arm (31.1 ± 8.4 min vs. 24.9 ± 6.9 min, respectively; *p* = 0.004) (Figure 1 and Figure 2). Postoperative pain assessed at 24 h using the visual analog scale (VAS) did not differ significantly between groups (VAS 3.20 ± 1.21 in the microdebrider group vs. 2.73 ± 1.30 in the coblation group; *p* = 0.191) (Figure 3).

**Figure 4:**
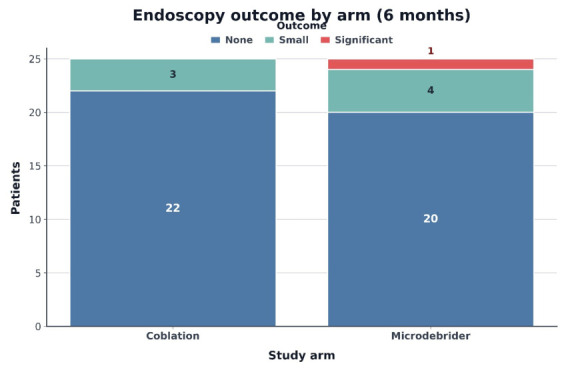
Comparison of endoscopy outcomes in both groups.

Early postoperative complications, including bleeding within 48 h, infection, halitosis, and neck pain, were infrequent and did not differ significantly between the two techniques (all *p* > 0.05) (Table 2). At 6-month endoscopic follow-up, the majority of patients demonstrated no residual adenoid tissue, observed in 80.0% (20/25) of the microdebrider group and 88.0% (22/25) of the coblation group. Only one patient, in the microdebrider arm, was classified as having significant residual tissue/recurrence. The three-category endoscopic outcome distribution did not differ significantly between groups (χ^2^ *p* ≈ 0.54), and comparison of dichotomous recurrence rates (1 vs. 0 events) yielded a Fisher’s exact test *p* value of 1.00 (Figure 4).

## 4. Discussion

Adenoid hypertrophy is a common pediatric condition and adenoidectomy remains one of the most frequently performed ENT procedures in children; over the last two decades the field has seen a proliferation of instrumented, endoscopic techniques (powered microdebriders, coblation/plasma ablation, suction diathermy and others) intended to improve visualization, reduce residual tissue and possibly decrease pain and bleeding compared with blind curettage [7,8]. The contemporary literature emphasizes that, while endoscopic approaches in general provide more controlled tissue removal under vision and therefore tend to lower residual adenoid tissue compared with blind curettage, the comparisons among the endoscopic options themselves (for example microdebrider versus coblation) are heterogeneous and do not show a single technique uniformly superior across all clinically important outcomes [3,7,8].

The present study compared transoral endoscopic-assisted adenoidectomy using a microdebrider (n = 25) to coblation (n = 25) and enrolled balanced groups with similar baseline characteristics (mean ages ~9.6 years, balanced sex distribution and comparable preoperative adenoid grade). The primary findings were a statistically significant difference in skin-to-skin operative time favoring the microdebrider (24.9 ± 6.9 min for microdebrider vs. 31.1 ± 8.4 min for coblation; *p* = 0.004), while early postoperative pain at 24 h (VAS) did not differ significantly (3.20 ± 1.21 vs. 2.73 ± 1.30; *p* = 0.191). Early adverse events (postoperative bleeding within 48 h, infection within 7 days, halitosis and neck pain) were low overall and did not differ between groups, and endoscopic control at six months showed high rates of no residual tissue in both arms (80% microdebrider, 88% coblation) with only one patient overall showing a clinically significant recurrence; these differences were not statistically significant. Of note, our times are longer than the 5–12 min ranges reported in some studies; differences in operative time definitions, workflow, and emphasis on meticulous hemostasis and endoscopic inspection likely account for much of this variation, in line with the heterogeneity described in recent reviews and network meta-analyses.

When these outcomes are compared with published studies and syntheses, several patterns emerge that help place our results in context. First, operative time is an outcome that consistently shows interstudy variability and depends heavily on device choice, an exact time definition, surgeon experience and case complexity. Some randomized trials and single-center reports have reported shorter operative times with coblation compared with the microdebrider [2,4], while other reports and pooled analyses indicate either no consistent difference or that microdebrider techniques can require less or more time depending on the study’s definitions and technical setup [7,8]. The network meta-analysis by Malas et al. [8] ranked techniques and found endoscopic microdebrider adenoidectomy to have a tendency toward longer estimated surgical time compared with some other techniques, but the authors emphasized a high degree of heterogeneity across trials and cautioned that differences in device models, power settings and surgical workflow influence these rankings. Thus, the finding of a longer coblation time is not contradictory to the literature; it is one plausible outcome among the possible inter-institutional patterns and most likely reflects the local device model, wand configuration, power settings and the team’s relative familiarity with the specific coblation system used. That interpretation is consistent with prior trial-level observations that exposure quality, wand ergonomics and the learning curve can materially alter measured operative time [3,4].

Second, early postoperative pain after adenoidectomy is low overall and small absolute differences are common but often of unclear clinical importance. Several reviews and randomized studies report that coblation may offer modest pain advantages compared with cold curettage but the advantage of coblation over the powered microdebrider is much less consistent [9]. Some single-center series observed lower pain scores and shorter pain duration after coblation compared with the microdebrider [4], while other studies and pooled analyses found no consistent difference between these two endoscopic methods or reported only small, transient differences dependent on analgesic regimens and measurement timings [10]. Juneja et al. [11] reported no clinically meaningful difference in postoperative discomfort between powered and conventional techniques, and Alaskarov et al. [10] similarly noted that early pain scores did not significantly differ between coblation and other modern techniques when standardized analgesic protocols were used. The absence of a significant difference in 24 h VAS pain scores in the present study is therefore concordant with prior randomized and prospective evidence, particularly given the single postoperative time point assessed.

Third, early complications after endoscopic-assisted adenoidectomy are generally infrequent and the available high-quality evidence finds no technique with a markedly superior safety profile across all adverse events. Large retrospective cohorts and observational series provide nuanced findings: for example, Liu et al. [2] analyzed 468 pediatric adenoidectomies and reported less intraoperative bleeding and shorter operative time associated with coblation, but also a higher incidence of certain adverse events (fever, neck pain, halitosis) in the coblation group compared with the microdebrider. Systematic reviews and network meta-analyses conclude that while some techniques may be favored for specific endpoints (e.g., less blood loss or fewer residual tissues) [7,8], no single approach is uniformly superior for all types of complications and in many pooled comparisons postoperative complication rates did not differ significantly. The neutral finding (no significant difference in bleeding, infection, and halitosis or neck pain) is therefore consistent with pooled evidence showing low absolute event rates and high between-study heterogeneity. It also underlines the practical reality that uncommon adverse events require much larger samples or registry-level data to detect modest technique-related differences.

Fourth, regarding completeness of resection and early recurrence, endoscopic approaches in general are associated with less residual adenoid tissue and lower revision rates compared with blind curettage; however, evidence directly comparing the microdebrider to coblation for long-term recurrence or clinically meaningful residual tissue is limited and mixed. Calvo-Henríquez et al.’s [3] review concluded that coblation adenoidectomy appears to offer better adenoid control compared with curettage and may reduce nasal obstruction, but it also emphasized the relative paucity of direct comparisons between coblation and the microdebrider and the need for standardized outcome definitions. With respect to completeness of resection and recurrence, endoscopic techniques consistently demonstrate advantages over blind curettage; however, evidence directly comparing coblation and the microdebrider for long-term outcomes remains limited. Juneja et al. [11] highlighted the superior endoscopic clearance achieved with powered adenoidectomy, and Abo Elmagd et al. [9] emphasized improved visualization and reduced residual tissue with microdebrider use. The high rates of ‘no residual’ adenoid tissue at six months in both groups (80% vs. 88%), together with the single clinically significant recurrence observed in the entire cohort, are consonant with broader findings that both techniques reliably achieve substantial tissue removal under endoscopic vision and that differences in short-term residual burden between them appear small in randomized comparisons.

Bringing these points together, the apparent conflicts among published trials and our data can be reconciled through a focus on effect modifiers that are repeatedly emphasized in the literature. Device-level factors (wand/handpiece design, blade geometry and power settings), intraoperative workflow (e.g., whether the wand needs intermittent cleaning or exchange), surgeon experience and learning-curve effects, case mix (adenoid size and whether concurrent procedures are performed), and precise definitions used for outcome measurement (how “operative time” is timed, single versus serial pain measures, and what constitutes “clinically relevant” residual tissue) all have strong explanatory power for why different centers report different relative advantages for coblation versus the microdebrider [3,7,8]. Several randomized trials and observational analyses explicitly attribute their differing operative-time results to exposure quality or to the specific coblation wand used [2,4]. Given that variability, the most defensible interpretation of our study is that both techniques are safe and effective in experienced hands, that the microdebrider produced shorter skin-to-skin time in your setting (likely reflecting local device/technique/experience factors), and that functional outcomes (pain, early complications, short-term residual tissue) were equivalent within the limits of the sample size.

Beyond clinical outcomes, the cost and availability of disposable devices are important determinants of technique selection. Coblation wands typically incur higher per-case costs than reusable microdebrider systems, and any advantages in bleeding or operative time must be balanced against these expenditures, particularly in resource-constrained settings. Our findings support an individualized approach in which surgeon experience, device availability, and cost considerations collectively guide the choice between these two endoscopic techniques.

It is important to acknowledge limitations that affect both the internal precision of the present trial and the ability to generalize these findings. The number of patients (25 per arm) provides adequate sensitivity for moderate-to-large differences in continuous variables such as operative time but is underpowered to detect small differences in pain or to identify infrequent adverse events; larger multi-center trials or pooled registry data are necessary to assess uncommon complications with confidence. Single-center execution and a small number of operating surgeons may introduce operator-specific effects; multi-center replication would help determine whether the microdebrider advantage in operative time persists when averaged across diverse workflows and device configurations. The 6-month follow-up window is useful for early residual assessment but longer follow-up (12–24 months) is preferable for evaluating late regrowth and true revision rates, which some studies suggest can continue to diverge over time [9,10,11]. Finally, reporting more device-level detail in methodology (specific coblation wand model and power setting, microdebrider blade type and rpm, exact definition of “skin-to-skin” time, analgesic regimen given intra- and post-operatively) would improve the ability to compare outcomes directly to other trials and meta-analyses; the literature continually emphasizes the value of standardized reporting for this reason [3,8].

Transnasal endoscopic approaches are also widely used and offer alternative routes of visualization and instrument access, particularly in smaller children or where transoral exposure is challenging. While network meta-analyses and single-center series indicate that endoscopic techniques in general improve visualization and reduce residual tissue compared with blind curettage, direct comparisons using the same device (e.g., microdebrider or coblation) remain sparse. Existing data suggest that both routes can achieve high clearance rates and low early recurrence when performed by experienced surgeons, but differences in instrumentation, learning curves, and patient selection may influence operative time, bleeding, and ease of access to and peritubal region. Our findings therefore apply primarily to the transoral route, and future studies comparing transoral and transnasal microdebrider and coblation adenoidectomy in parallel would be valuable to determine whether the visualization and postoperative recovery differ significantly between access routes.

In summary, the present randomized comparison shows that both microdebrider and coblation endoscopic adenoidectomy are safe and effective, with comparable early pain, early complication rates and six-month residual/recurrence outcomes; a significantly shorter operative time for the microdebrider in our cohort appears plausibly related to institution- and device-specific factors. These findings align with systematic reviews and network meta-analyses that find endoscopic techniques outperform blind curettage for completeness of resection but do not demonstrate a single superior endoscopic technique across all outcomes [9,10,11]. In practice, device availability, cost and surgeon experience remain reasonable criteria to guide technique selection, while future research should prioritize larger, multi-center RCTs with standardized device reporting and longer follow-up to determine whether small differences in time or short-term pain translate into material clinical or economic benefits.

## 5. Conclusions

In this study, coblation endoscopic adenoidectomy and microdebrider adenoidectomy were both safe and successful. In our setting, the microdebrider was linked to a shorter operating time, but the techniques’ six-month endoscopic residual/recurrence, short-term complications, and early pain were comparable.

Device-specific factors, surgeon experience and procedural definitions likely influence operative time and may explain variation across studies; larger multi-center trials with standardized device reporting and longer follow-up are warranted.

## Figures and Tables

**Table 1:** Baseline characteristics.

Variable	Microdebrider (n = 25)	Coblation (n = 25)	*p*-Value
Age (years)	9.56 ± 3.99	9.64 ± 3.84	0.977
Weight (kg)	29.06 ± 9.88	30.05 ± 9.91	0.725
Sex (M; F)	14 (56%); 11 (44%)	18 (72%); 7 (28%)	0.377
Primary indication (top categories shown)	Nasal obstruction 11 (44%); obstructive sleep symptoms 6 (24%); recurrent OM 6 (24%)	Nasal obstruction 14 (56%); recurrent OM 8 (32%); obstructive sleep 1 (4%)	0.096
Preoperative adenoid grade (2/3/4)	3:13 (52%); 2:7 (28%); 4:5 (20%)	3:15 (60%); 2:7 (28%); 4:3 (12%)	0.704

**Table 2:** Short-term complications and events.

Complication	Microdebrider (n = 25)	Coblation (n = 25)	*p*-Value
Post-op bleed within 48 h	1 (4%)	2 (8%)	0.55
Infection within 7 d	1 (4%)	1 (4%)	1
Halitosis at 24 h	1 (4%)	2 (8%)	0.55
Neck pain	1 (4%)	2 (8%)	0.55
Total	4 (16%)	7 (28%)	0.32

## Data Availability

The datasets generated and/or analyzed during the current study are not publicly available due to regulatory policy but are available from the corresponding author on reasonable request.

## References

[1] Sjogren P.P., Thomas A.J., Hunter B.N., Butterfield J., Gale C., Meier J.D. (2018). Comparison of pediatric adenoidectomy techniques. Laryngoscope.

[2] Liu T., Ji C., Sun Y., Bai W. (2022). Adverse events of coblation or microdebrider in pediatric adenoidectomy: A retrospective analysis in 468 patients. Laryngoscope Investig. Otolaryngol..

[3] Calvo-Henriquez C., Rueda-Fernandez-Rueda M., Garcia-Lliberos A., Maldonado-Alvarado B., Mota-Rojas X., Maniaci A., Iannella G., Jimenez-Huerta I. (2023). Coblator adenoidectomy in pediatric patients: A state-of-the-art review. Eur. Arch. Otorhinolaryngol..

[4] Mularczyk C., Walner D.L., Hamming K.K. (2018). Coblation versus microdebrider in pediatric adenoidectomy. Int. J. Pediatr. Otorhinolaryngol..

[5] Singh J., Bhardwaj B. (2020). The comparison between microdebrider assisted adenoidectomy and coblation adenoidectomy: Analyzing the intraoperative parameters and post-operative recovery. Indian J. Otolaryngol. Head Neck Surg..

[6] Metwally M., Omran T., Abdel-Maksoud M., El-Malt A. (2023). Assessment of outcomes of endoscopic-assisted adenoidectomy: Microdebrider versus coblation in children. Zagazig Univ. Med. J..

[7] Sun Y.L., Yuan B., Kong F. (2023). Comparison between different approaches applied in pediatric adenoidectomy: A network meta-analysis. Ann. Otol. Rhinol. Laryngol..

[8] Malas M., Althobaiti A.A., Sindi A., Bukhari A.F., Zawawi F. (2023). Comparison of the efficacy and safety of conventional curettage adenoidectomy with those of other adenoidectomy surgical techniques: A systematic review and network meta-analysis. J. Otolaryngol. Head Neck Surg..

[9] Abo Elmagd E.A., Khalifa M.S., Abeskharoon B.K., El Tahan A.A. (2021). Comparative study between conventional adenoidectomy and adenoidectomy using microdebrider. Egypt. J. Otolaryngol..

[10] Alaskarov E. (2024). Comparison of classical, coblation, and combined adenoidectomy techniques in pediatric patients: A single-blind, prospective study. Eur. Arch. Otorhinolaryngol..

[11] Juneja R., Meher R., Raj A., Rathore P., Wadhwa V., Arora N. (2019). Endoscopic assisted powered adenoidectomy versus conventional adenoidectomy: A randomised controlled trial. J. Laryngol. Otol..

